# Reliability and validity test of the Chinese version of the spiritual care-giving scale in nursing undergraduates and its application

**DOI:** 10.12669/pjms.40.10.9021

**Published:** 2024-11

**Authors:** Ying Huang, Dan Lv, Tao Wang, Haoran Zhang, Lei Zhang

**Affiliations:** 1 Ying Huang, Department of Nursing, Chengde Medical University, Chengde 067000, Hebei, China; 2 Dan Lv, Department of Cardiac Medicine, Affiliated Hospital of Chengde Medical University, Chengde 067000, Hebei, China; 3 Tao Wang, Department of Urinary Surgery, Affiliated Hospital of Chengde Medical University, Chengde, 067000, Hebei, China; 4 Haoran Zhang, Department of Cardiac Medicine, Affiliated Hospital of Chengde Medical University, Chengde 067000, Hebei, China; 5 Lei Zhang, Department of Nursing, Chengde Medical University, Chengde 067000, Hebei, China

**Keywords:** Nursing Undergraduates, Spiritual Cognition, Reliability, Validity

## Abstract

**Objective:**

To evaluate the reliability and validity of the Chinese Version of the Spiritual Care-giving Scale (C-SCGS) in nursing undergraduates and its application.

**Methods:**

This was a retrospective study. A questionnaire survey was conducted among 262 senior nursing undergraduates from Chengde Medical University, China from May to June, 2023 through convenience sampling method. The C-SCGS and the general demographic data table were employed to investigate the subjects.

**Results:**

The C-SCGS retained 34 items, and four common factors were extracted by exploratory factor analysis, with a cumulative contribution rate of 65.15%. The I-CVI value of the scale was 0.88-1.0, and the S-CVI/Ave value was 0.94. The Cronbach's α coefficient of the total scale was 0.957, that of each dimension ranged from 0.850 to 0.944, with a split-half coefficient of 0.887.

**Conclusion:**

The C-SCGS may be used to evaluate the spiritual cognition of Chinese nursing undergraduates by virtue of its good reliability and validity.

## INTRODUCTION

Spirituality, as a kind of subjective feeling and internal experience of human beings, is a spiritual force that is intrinsically related to the meaning of existence.^1^ Spirituality care constitutes a crucial part of palliative care and holistic care, which is markedly related to the quality of life of patients^2^-^4^. Nursing students are the preparatory army of future clinical nurses. Their awareness of spirituality and identification of spiritual care needs play a vital role in solving spiritual problems of patients and their families in the future.^5^,^6^

Spirituality is an abstract, multidimensional and complex concept, which has not been included in the unified curriculum in nursing education in China. As a result, the majority of Chinese nurses are not very familiar with the concept of spirituality.^5^ Foreign scholars have developed some assessment tools to evaluate nurses’ views on spiritual care, such as Spirituality and Spiritual Care^7^ and Spiritual Care-Giving Scale (SCGS). SCGS was developed by Tiew et al.^8^ in 2012 to measure Singapore nursing students’ cognition of spirituality or spirituality care, and to test whether they are suitable for the clinical practice of spirituality care. It was later applied to nurses to test their reliability and validity.^9^ Chinese scholar Hu Yanli et al.^10^ introduced and localized the Chinese version - Spiritual Care Giving Scale (C-SCGS) and applied it to clinical nurses in China. Finally, it is proved that the Chinese version of the scale has good reliability and validity. However, given the huge differences in clinical practice between clinical nurses and nursing students, it is unknown whether the C-SCGS can be used to measure the spiritual cognition of nursing students in China. In view of this, this study was conducted to evaluate the validity and reliability of the C-SCGS in nursing students in China and provide a reference for their spiritual care education.

## METHODS

This was a retrospective study. A questionnaire survey was conducted among 262 senior nursing undergraduates from Chengde Medical University from May 2019 to June, 2023 through a convenience sampling method. The sample size was estimated by using the principle of determining the sample size of the influencing factors research, and 5-10 times the number of variables was taken as the final sample size of the investigation. The number of variables investigated in this study was 34. According to the standard of 1:5 and taking into account 20% loss, the sample size of the study was at least 204 nursing undergraduates. Therefore, a total of 251 nursing undergraduates were surveyed in this study.

### Ethical Approval:

The study was approved by the Institutional Ethics Committee of Chengde Medical University (No.: CYFYLL2018003; January 01, 2018), and written informed consent was obtained from all participants.

### Inclusion criteria:

- Full-time nursing undergraduates;
- Senior students who have completed a one-year clinical internship (currently in the stage of returning to school and waiting for employment);
- Nursing undergraduate students who informed and agreed to participate in this study.

### Exclusion criteria:

- Based on the principle of information saturation, data collection was ceased if no new themes emerged from the data analysis.

### Study tools:

(1) General data questionnaire: designed by the investigators themselves, mainly including the gender, age, grade and degree of understanding of spirituality of nursing undergraduates. (2) The Chinese version - Spiritual Care Giving Scale (C-SCGS) was introduced and translated by Hu Yanli et al. of Jilin University in China in October 2018.^10^ The scale includes four dimensions, namely, spirituality care characteristics, spirituality and spirituality care definition, spiritual cognition, spirituality and spirituality care value, with a total of 34 items. The scale adopts Likert 6-level scoring method, and assigns 1-6 points from “strongly disagree” to “strongly agree”. The total score of the scale is 34 to 206 points. The higher the score, the more positive the nurses are about spirituality and spirituality care. Cronbach’s α coefficient of each dimension was 0.836-0.941, that of spirituality care characteristics was 0.941, that of spirituality and spirituality care definition was 0.852, that of spiritual cognition was 0.836, and that of spirituality and spirituality care value was 0.866. The split-half reliability coefficient was 0.893. The scale has good reliability and validity.^11^

After the Chinese version - Spiritual Care Giving Scale (C-SCGS) was authorized by the author and approved by the ethics committee, the subjects were selected in strict accordance with the inclusion and exclusion criteria. Then, the C-SCGS was used to evaluate the spiritual cognition of nursing undergraduates. In April 2023, 20 nursing undergraduates were selected from a university for a pre-survey. The investigators explained the purpose and significance of this study to the subjects by using unified instructions and signed an informed consent form to discuss with each subject their understanding of the description of the scale, each item and the way of answering. The results showed that the subjects could understand the meaning of each item well, and did not propose any amendments, and the filling time was between two and five minutes. After that, a questionnaire survey was conducted among nursing undergraduates in a school, and the subjects were asked to fill it out independently in an anonymous way and collect it on the spot. A total of 262 questionnaires were distributed, with a recovery rate of 100%, of which 251 were valid, with an effective rate of 95.80%.

### Statistical analysis

All data in this study were statistically analyzed using SPSS 25.0. For the survey results of the general data of subjects, the counting data were expressed as percentages, and the measurement data were expressed as mean ± standard deviation, an independent sample t test is used in this study. The reliability and validity test of the scale was carried out as follows.

### Item analysis:

the total score of the scale was sorted in descending order by the critical ratio method, with the top 27% of the subjects representing the high score and the last 27% representing the low score. An Independent sample t test was conducted on each item, and items with no statistical difference between the two groups or with a T critical value less than three were deleted.^12^ The correlation coefficient between the scores of each item and the total score of the scale was calculated by the method of the total correlation coefficient, and the items with a correlation coefficient less than 0.4 and not reaching a significant level were deleted.^12^ Cronbach’s α coefficient was used for evaluation. If Cronbach’s α coefficient increases greatly after deleting an item, it is considered that the attribute to be measured in this item is different from other items, so this item should be deleted. If item commonality < 0.2 and factor load value < 0.45, consider deleting the item.^12^

### Validity test:

the content validity test evaluation form collected expert evaluation opinions and suggestions. A 4-level scoring method was adopted for each item, with:

- indicating “strongly irrelevant”,
- indicating “must be modified, otherwise not relevant”,
- indicating “strongly correlated, but a small amount of modification is required”, and
- indicating “strongly correlated, according to the expert evaluation results”.

The scales I-CVI and S-CVI/Ave were calculated respectively. I-CVI was calculated by dividing the number of experts whose evaluation results were three or four by the total number of experts, and S-CVI/Ave was the average of all I-CVI.^13^ Exploratory factor analysis was used for structural validity: the extracted eigenvalues were > 1; cumulative contribution rate > 50%; factor load > 0.4; At the same time, if the load of two or more factors is greater than 0.4 and the difference is less than 0.2, the items should be deleted.^13^ Cronbach’s α coefficient and split-half reliability were used as detection indexes in the reliability test.

## RESULTS

All 251 senior nursing undergraduate students included were aged between 21 and 25 years old, with an average age of (22.85±1.01) years; Among them, 6.8% were 21 years old, 32.3% were 22 years old, 36.7% were 23 years old, 17.5% were 24 years old and 6.8% were 25 years old; Moreover, 93.2% were female and 6.8% were male. 62.9% of nursing students reported that they knew something about spirituality, and 37.1% of nursing students knew nothing about it. All nursing students have completed their one-year clinical internship in their senior year.

The critical ratio of the scale ranged from 7.67 to 12.92, the total correlation coefficient was 0.469-0.764, and the commonality of the items was 0.490-0.754. All the items in the scale were retained for exploratory factor analysis.

**Fig.1:**
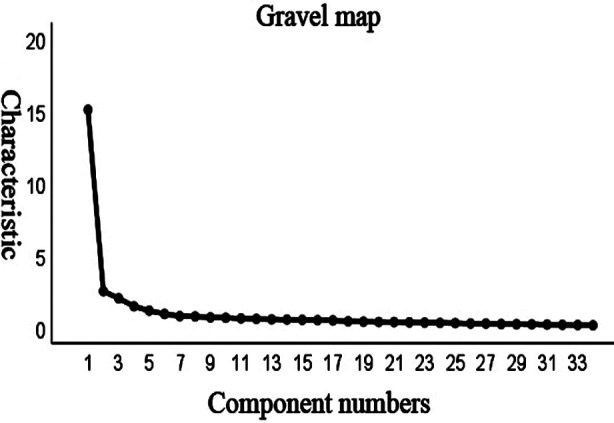
Scree plot results of the C-SCGS scale.

KMO and Bartlett tests were conducted on the data of 251 nursing students. The results showed that the KMO value in the total table was 0.944, and the Bartlett spherical test was statistically significant (χ^2^ = 5963.946, *P* < 0.001). See Table-I for the analysis results of each factor and Fig.1 for the scree plot.

**Table-I:** Factor loading matrix results of exploratory factor analysis of the C-SCGS scale.

Item	Factor				

	1	2	3	4	5
1. Everyone has spirituality (mind)	0.092	0.650	0.003	0.239	0.051
2. Spirituality constitutes a crucial part of human beings	0.128	0.776	0.036	0.208	0.019
3. Spirituality is a part of power that can make people reach an agreement in peace/tranquility and harmony	0.201	0.743	0.103	0.189	0.219
4. Spirituality is an internal emotional expression that affects people’s behaviour	0.285	0.735	0.080	0.121	0.257
5. Spirituality is a part of our heart	0.176	0.743	0.195	0.024	0.182
7. The happiness of the soul is of great importance to the individual’s emotional health	0.257	0.617	0.026	0.299	0.314
8. Spirituality drives individuals to find answers to the meaning and purpose of life.	0.373	0.537	0.173	0.079	0.392
6. Spirituality refers to finding the meaning of good and bad events in life.	0.225	0.248	0.176	0.112	0.628
9. A person without spirituality is not a complete person	0.059	0.153	0.271	0.027	0.728
10. Spiritual needs are met through one’s connection with others, greater energy or nature	0.276	0.331	-0.099	0.369	0.602
13. Good nursing itself is spirituality care	0.314	0.393	0.475	0.221	-0.043
15. Spirituality care means respecting patients’ religious or personal beliefs	0.237	0.052	0.694	0.283	0.052
16. Sensitivity and intuition help nurses provide spirituality care	0.373	0.234	0.445	0.413	0.150
17. One of the forms of spirituality care is to be with patients	0.213	0.117	0.478	0.435	0.380
20. Spirituality care enables patients to find the meaning and purpose of their illness	0.153	0.083	0.680	0.087	0.410
21. Spirituality care is to support or help patients maintain their religious beliefs	0.199	0.001	0.778	0.085	0.136
11. Spirituality care constitutes a crucial part of holistic nursing	0.294	0.343	0.180	0.622	0.232
12. Spirituality care is not just religious care	0.349	0.454	0.119	0.634	0.033
14. Spirituality care is a process, not a one-time event or activity	0.496	0.412	0.221	0.510	-0.033
18. Nurses provide spirituality care for patients by respecting their religious and cultural beliefs	0.199	0.244	0.450	0.630	0.091
19. Nurses give patients enough time to talk about and explore their fears, anxieties and troubles and provide them with spirituality care	0.255	0.224	0.321	0.554	0.260
22. I comfortably provide spirituality care for patients	0.599	0.164	0.391	-0.047	0.154
23. Nurses provide spirituality care for patients by respecting their dignity	0.488	0.410	0.395	0.044	-0.192
24. Spirituality care should take into account the patient’s spiritual concept.	0.538	0.337	0.246	0.310	0.007
25. Nurses with spiritual awareness are more likely to provide spirituality care.	0.665	0.235	0.283	0.086	0.241
26. Spirituality care needs to be aware of one’s own spiritual world	0.690	0.113	0.133	0.198	0.398
27. The concept of spirituality care should be integrated into nursing education curriculum	0.774	0.112	0.296	0.044	0.126
28. Spirituality care should be actively strengthened in nursing practice	0.805	0.158	0.257	0.074	0.087
29. The ability to provide spirituality care is developed through experience	0.782	0.205	0.202	0.244	0.021
30. Spirituality care is valuable for giving patients hope	0.759	0.190	0.161	0.180	0.180
31. Spirituality (mind) is influenced by personal life experiences	0.727	0.142	-0.031	0.343	0.161
32. Spirituality (mind) helps/helps to face the difficulties and problems in life	0.757	0.195	0.058	0.319	0.174
33. Providing spirituality care for patients requires a nurse-patient relationship based on trust	0.679	0.283	0.035	0.386	0.019
34. Providing spirituality care to patients as a team is important	0.513	0.340	0.144	0.360	0.126

### Content validity:

In this study, ten nursing experts were invited to evaluate the content validity of the C-SCGS. The results showed that the I-CVI value was 0.88-1.0 and the S-CVI/Ave value was 0.94, demonstrating good content validity. Reliability analysis results: The results showed that Cronbach’s α of the total scale was 0.957, indicating that the C-SCGS scale has good internal consistency when applied to nursing students. Table-II

**Table-II:** Reliability analysis of C-SCGS(n=251).

	Spirituality care characteristics	Spirituality and spirituality care definition	Spiritual cognition	Spirituality and spirituality care value	Total scale score
Cronbach’s α coefficient	0.944	0.860	0.856	0.850	0.957
Split-half reliability	0.905	0.862	0.798	0.743	0.887

It was shown in this study that the total score of spiritual cognition of Chinese nursing undergraduates was (161.37±19.53), among which the score of spirituality care characteristics was (63.17 ± 8.15), that of spirituality and spirituality care definition was (36.51 ± 5.61), that of spiritual cognition was (24.18 ± 3.48). and that of spirituality and spirituality care was (37.51 ± 5.17). Table-III

**Table-III:** C-SCGS scores(n=251).

	Mean ± standard deviation	Median (p25, p75)	Minimum value	Maximum value	Total points
Spirituality care characteristics	63.17±8.15	65(60,66)	28	78	78
Spirituality and spirituality care definition	36.51±5.61	37(32,40)	20	48	48
Spiritual cognition	24.18±3.48	25(23,26)	6	30	30
Spirituality and spirituality care value	37.51±5.17	39(34,40)	17	48	48

Total score	161.37±19.53	166(151,170)	95	204	204

## DISCUSSION

In this study, the C-SCGS scale was used to test the reliability and validity of Chinese nursing undergraduates. The results show that the scale has good reliability and validity in such a population, and the items in the C-SCGS scale are easy to understand, with an average time of three minutes and thirty seconds, showing good feasibility and operability. Meanwhile, the effective response to questionnaire was as high as 95.80%, which shows that the scale is feasible and can be used to measure the spiritual cognition of Chinese nursing undergraduates.

Validity refers to the degree to which a research tool reflects its expected research concept. The higher the degree to which it reflects the expected research concept, the better its validity.^12^,^13^ In this study, content validity and structural validity are utilized to evaluate the scale. Content validity is to test the appropriateness and representativeness of the items in the scale.^14^

It is considered that the Cronbach’s α coefficient of the total scale is above 0.80, which means good reliability of the scale. If it is above 0.90, it means “very good” reliability.^15^ For the Cronbach’s α coefficient of the sub-scale, above 0.7 means good reliability. The results of this study show that the Cronbach’s α coefficient of the C-SCGS scale is between 0.850 and 0.944, and the split-half coefficient is 0.887, demonstrating good reliability of the scale when applied to undergraduate nursing students. The results are consistent with those of the source scale (Cronbach’s α coefficient ranges from 0.836 to 0.941, and the split-half coefficient is 0.893), indicating the availability of the C-SCGS scale for nursing undergraduates in China.

It is shown in this study that the score of spiritual cognition of Chinese nursing undergraduates is high, which is consistent with the results of foreign scholar Chan et al.^16^ on the spiritual cognition of nursing undergraduates. However, it is worth noting that although the senior nursing students in this study have a high level of spiritual cognition, nearly 37.1% of nursing students know nothing about the spiritual related content. The reason may be related to the fact that spiritual related education has not been offered in colleges and universities and clinical practice in China.^17^,^18^ Most colleges and universities in China have not offered spiritual education courses, and most hospitals have not included spiritual education and other related contents in their clinical practice. It can be seen that, on the whole, the spiritual cognition level of nursing students in China is not ideal. Research shows that if nurses have a high level of spiritual cognition, it will be beneficial for them to carry out better spiritual care for patients. This also suggests that when facing senior nursing undergraduates who are about to work in clinical positions, spiritual awareness education should be actively provided to them to equip them with spiritual knowledge, so that they can better solve the spiritual needs of patients.

### Limitations

Only nursing undergraduates from one university were included as subjects, and all are seniors, resulting in a relatively small sample size. The sampling scope should be expanded in subsequent studies and nursing students with different academic qualifications and grades should be included to further test the reliability and validity of the scale.

## CONCLUSIONS

Based on the item analysis, validity analysis and reliability analysis of the C-SCGS scale in this study. This scale shows good reliability and validity when applied to nursing undergraduates. The scale items are concise, clear and easy to understand, and may be used as an assessment tool to evaluate the spiritual cognition of Chinese nursing undergraduates. The test of this scale in nursing undergraduates might provide a powerful tool for understanding the spiritual cognition of nursing undergraduates.

### Authors’ Contributions:

**YH and DL** carried out the studies, participated in collecting data, and drafted the manuscript.

**TW and HZ** performed the statistical analysis and participated in its design.

**LZ** performed the statistical analysis and participated in its design; All authors read and approved the final manuscript, are responsible and accountable for the accuracy or integrity of the work.
